# Number of glioma polyploid giant cancer cells (PGCCs) associated with vasculogenic mimicry formation and tumor grade in human glioma

**DOI:** 10.1186/1756-9966-32-75

**Published:** 2013-10-15

**Authors:** Yang Qu, Li Zhang, Zhe Rong, Tao He, Sai Zhang

**Affiliations:** 1 Department of Neurosurgery, Logistic University Affiliated Hospital, Logistic University of Chinese People’s Armed Police Force, Tianjin 300162, P.R China; 2 Graduate School of Tianjin University of Traditional Chinese Medicine, Tianjin 300193, P.R China; 3 Department of Pathology, Tianjin Union Medicine Center (Nankai University Affiliated Hospital), Tianjin, P.R China; 4 Department of Basic Medicine & Experimental Technology, Division of Clinical Medicine, Logistic University of Chinese People’s Armed Police Force, Tianjin 300162, P.R China; 5 Department of pathology, Logistic University Affiliated Hospital, Logistic University of Chinese People’s Armed Police Force, Tianjin 300162, P.R China

**Keywords:** Polyploid giant cancer cells, Stem cells, Gliomas, Vasculogenic mimicry, Mosaic vessel

## Abstract

**Background:**

Polyploid giant cancer cells (PGCCs) contribute to solid tumor heterogeneity. This study investigated the relationships among PGCCs numbers, vasculogenic mimicry (VM) formation, and tumor grades in glioma.

**Methods:**

A total of 76 paraffin-embedded glioma tissue samples, including 28 cases of low grade and 48 cases of high grade gliomas, were performed with H&E and immunohistochemical staining for Ki-67 and hemoglobin. The size of PGCCs nuclei was measured by a micrometer using H&E section and defined as at least three times larger than the nuclei of regular diploid cancer cells. The number of PGCCs and different blood supply patterns were compared in different grade gliomas. Microcirculation patterns in tumors were assessed using CD31 immunohistochemical and PAS histochemical double staining. Human glioma cancer cell line C6 was injected into the chicken embryonating eggs to form xenografts, which was used to observe the PGCCs and microcirculation patterns.

**Results:**

In human glioma, the number of PGCCs increased with the grade of tumors (*χ*^2^ = 4.781, *P* = 0.015). There were three kinds of microcirculation pattern in human glioma including VM, mosaic vessel (MV) and endothelium dependent vessel. PGCCs were able to generate erythrocytes via budding to form VM. The walls of VM were positive (or negative) for PAS staining and negative for CD31 staining. There were more VM and MVs in high grade gliomas than those in low grade gliomas. The differences have statistical significances for VM (*t* = 3.745, *P* = 0.000) and MVs (*t* = 4.789, *P* = 0.000). PGCCs, VM and MVs can also be observed in C6 chicken embryonating eggs xenografts.

**Conclusions:**

The data demonstrated presence of PGCCs, VM and MVs in glioma and PGCCs generating erythrocytes contribute the formation of VM and MVs.

## Background

Polyploid giant cancer cells (PGCCs) refer to the special sub-population of cancer cells [1,2] and usually have increased cell size with single giant nuclei or multinuclei with significant variation in shape, chromatin pattern, and number of nuclei. The PGCCs are the most commonly described histopathology features of human tumors, particularly in high grade and advanced stage tumor and usually correlate with poor prognosis [3-5]. PGCCs have often been considered an intermediate product of genomic instability [6-10], although the mechanisms of the PGCCs formation and their function in the development of human cancer are largely undefined. PGCCs remarkably differ from regular diploid cancer cells in morphology, size, chromosomal abnormalities, tumorigenic ability, radioresistance and chemoresistance. Indeed, these cells may contribute to tumor maintenance and recurrence. Zhang et al. reported that PGCCs had remarkable biologic features of cancer stem cells [11,12]. PGCCs could form through endoreduplication or cell fusion. PGCCs divided asymmetrically and cycled slowly, contributed to the heterogeneous tumor growth and drug resistance, which can be considered as the seed cells fueling the growth and recurrence of human cancer. Furthermore, the number of PGCCs varies with the malignant grade of tumor. There are more PGCCs in malignant tumor than those in benign, in high grade tumor than those in low grade tumor [11].

Angiogenesis is the physiological process involving the growth of new blood vessels from pre-existing blood vessels. Angiogenesis is also a vital process in embryonic development, wound healing, and carcinogenesis. Cancer development usually undergoes an initial period of avascular growth followed by vasculogenic mimicry (VM) and mosaic vessels (MVs) that connect with endothelium dependent vessels to obtain sufficient blood and oxygen supply to support tumor cell growth, invasion, and metastasis [13,14]. More aggressive tumors require more blood supply to support their rapid cell growth than that in the low grade tumors. VM has increasingly been recognized as a pattern of angiogenesis. Accumulating evidences have demonstrated that high grade malignant tumors including inflammatory breast cancer [15], prostate cancer [16], and invasive ovarian cancer [17], sarcoma [18,19], and hepatocellular carcinoma [14] utilize VM to support tumor cell growth, invasion and metastasis. Erythrocytes carry oxygen to tissues and cells, and bone marrow is generally considered the main source of erythrocytes. However, Zhang et al. showed that cobalt chloride (CoCl_2_) treatment of HEY, SKOv3, BT-549 and MDA-MB-231 cells was able to form PGCCs, express the stem cell markers, and induce generation of erythrocytes expressing different forms of hemoglobin both in vitro and in vivo [20]. Since tumor cells can generate erythrocytes, it is no doubt that tumor cells and their generating erythrocytes can form VM structure during tumor development and progression.

High grade malignant glioma is one of the leading causes of cancer death in many countries and the prognosis is very poor [21,22]. Therefore, in this study, we determined whether VM and PGCCs are present in human gliomas and then associate with tumor grade, and whether PGCCs-generated erythrocytes contributed the formation of VM and MVs.

## Methods

### Tissue samples

A total of 76 paraffin-embedded glioma tissues were obtained from the Tumor Tissue Bank of Tianjin Union Medicine Center and Logistic University of Chinese People's Armed Police Force. The patients underwent surgery between 1995 and 2009 and the diagnosis was verified by pathologists. These patients included 42 males and 34 females and were histologically divided into two groups, 28 cases of low grade gliomas (grade I and II with the mean age of 32.47 ± 1.97) and 48 cases of high grade gliomas (grade III and IV with the mean age of 50.41 ± 1.89) according to the World Health Organization (WHO) classification based on the morphology and Ki-67 immunohistochemical staining. This study was approved by the institutional research committee and the confidentiality of patients’ information has been maintained.

### Immunohistochemical (IHC) and histochemical double-staining

To confirm the identity of the cells lining the walls and whether VM was present in the tissues, formalin-fixed and paraffin-embedded tissues were cut at 4 μm, dried for 2 h at 60°C and then deparaffinized in xylene and rehydrated in a series of alcohol. Subsequently, heat-induced epitope retrieval was achieved in 0.01 M citric acid buffer (pH = 6.0) in a microwave oven and endogenous peroxidase activity was blocked with 3% hydrogen peroxide for 10 min. The primary monoclonal mouse anti-CD31 (MAB-0031, Maixin.Bio, Fujian, China), Ki-67 (MAB-0672, Maixin.Bio, Fujian, China) and goat polyclonal anti-hemoglobin-β/γ/ϵ/δ chain (Santa Cruz Biotechnology Inc. sc-22718)antibodies were used at a dilution of 1:100. The MaxVision™/HRP (Maixin.Bio) was used. Visualization was performed using the diaminobenzidine method (Maixin.Bio).

### Review of scoring Ki-67 stained tissue sections and glioma grading

Tumor cells with brown nuclei were considered positive. We reviewed five fields per section at 400× magnification and positive cells were counted in 100 tumor cells for each field. The mean percentage of positive cells was used to assess the grading of gliomas.

### Assessment and quantification of different blood supply patterns

Five microscopic fields in each tissue section were reviewed under microscopy with × 400 magnification and the average was considered as the number of different blood supply patterns. Endothelial dependent vessels (EVs) counting standard: According to the standard introduced by Weidner et al. [23,24], capillary vessels and microvessels in the tumor stained with CD31 were counted. A single positively stained endothelial cell can be counted as one EV. VM counting standard: The wall of VM is lined with tumor cells, and red cells can be found in the VM, without inflammation cells or red cell leakage around the VM [25]. MVs counting standard: The vessel wall was lined with both tumor and endothelial cells [14,25].

### PGCCs counting and definition

Five microscopic fields in each tissue section were reviewed and scored under microscopy with × 400 magnification and the average was summarized. The size of PGCCs nuclei was measured by a micrometer using H&E section. We defined the PGCC as a cancer cell that the nucleus of PGCC is at least three times larger than that of diploid cancer cell according to Zhang et al. description [11].

### Tumor xenografts in chicken embryonating eggs

Fresh fertilized eggs (less than 5 days after fertilization) (Tianjin Shengchi Inc.) were kept under 75% humidity and 37°C. At day 3 after incubation, the egg shell was cleaned with 75% ethanol. A square window (1 × 1 mm^2^) was opened in the end of air cell. The shell was removed and 0.1 ml PBS with 5 × 10^6^ glioma C6 cells was injected into the chorioallantoic membrane (CAM) of each egg. The opening was then closed with a cellophane tape and the eggs were incubated until the 20^th^ day. All these operations were performed in the sterile environment. These fertilized eggs were rotated with 45 degrees every day and the air cell end was always kept upright. At day 20 after incubation, the fertilized eggs were put into the -20°C freezer to kill the chicken embryos and then the tumor mass were dissociated. The tumor tissues were fixed with formalin and embedded with paraffin for H&E staining to observe the structure of different blood supply patterns and erythrocytes generated by PGCCs.

### Statistical analysis

The statistical analysis was performed using SPSS statistical analysis software (SPSS, Chicago, IL). An unpaired *t*-test was performed to analyze the differences in the number of VM, MVs and EVs. The *χ*^2^ test was used for the PGCCs number comparison among different grades of gliomas. A *P*-value less than 0.05 was considered statistically significance.

## Results

### Number of PGCCs associated with histologic grade of gliomas

**Figure 1:**
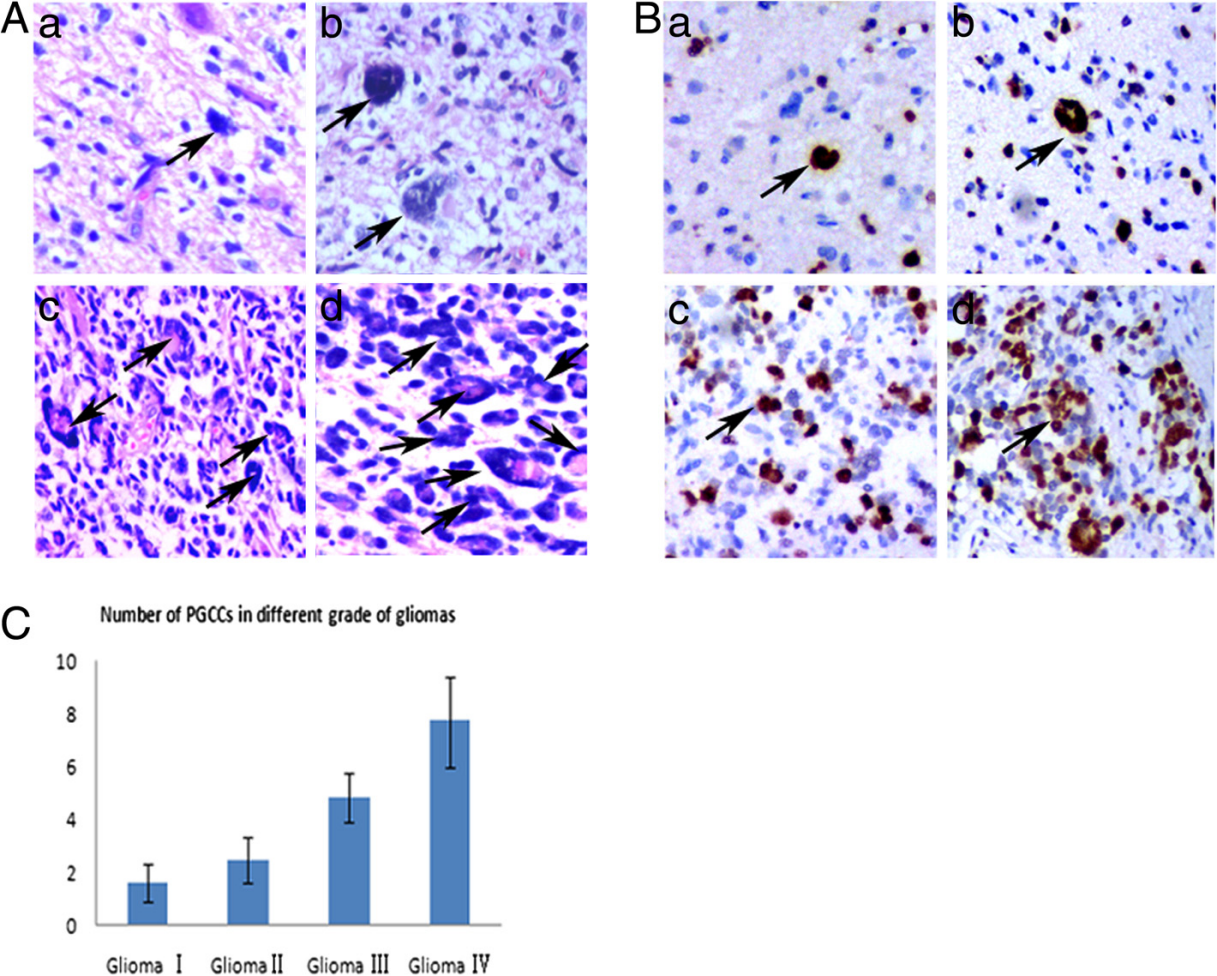
**Identification of PGCCs in glioma tissues. A**. PGCCs present in human gliomas. **a)** PGCCs in grade I gliomas (Black arrow points) (×200). **b)** PGCCs in grade II gliomas (Black arrows point) (×200). **c)** PGCCs in grade III gliomas (Black arrows point) (×200). **d)** PGCCs in grade IV gliomas (Black arrows point) (×200). **B**. Ki-67 IHC staining in gliomas and black arrows indicate the PGCCs. **a)** Ki-67 expression in grade I gliomas (×200). **b)** Ki-67 expression in grade II gliomas (×200). **c)** Ki-67 expression in grade III gliomas (×200). **d)** Ki-67 expression in grades IV gliomas (×200). **C**. Association of PGCCs number with the grades of human gliomas.

To grade all these 76 cases of glioma, new sections were cut from 76 paraffin-embedded glioma samples and stained with H&E and immunohistochemistry for further analysis. These tumors were graded by two pathologists according to the morphologic characteristics and Ki-67 IHC staining. Results of micrometer measure and morphologic observation showed presence of PGCCs in glioma tissues with giant or multi nuclei (Figure 1A). The size of PGCC nucleus was three times and up to 10–20 times larger than that of the regular diploid cancer cell. The shape of PGCCs nuclei was irregular. Ki-67 IHC staining data showed that Ki-67 expressed in all the glioma tissues and the positive ratio increased with the grade of gliomas. Most of PGCCs were positive for Ki-67 staining (Figure 1B). Based on these morphologic characteristics and Ki-67 staining, 76 cases of glioma were graded into 28 cases of low grade glioma (4 cases of grade I and 24 cases of grade II) and 48 cases of high grade (28 cases of grade III and 20 cases of grade IV). PGCCs can be observed in all these glioma tissues (Figure 1A), but there were more PGCCs in high grade tumors than those in low grade tumors and the difference was statistically significant (*χ*^2^ = 4.781, *P* = 0.015) (Figure 1C).

### Erythrocyte generation by PGCCs

**Figure 2:**
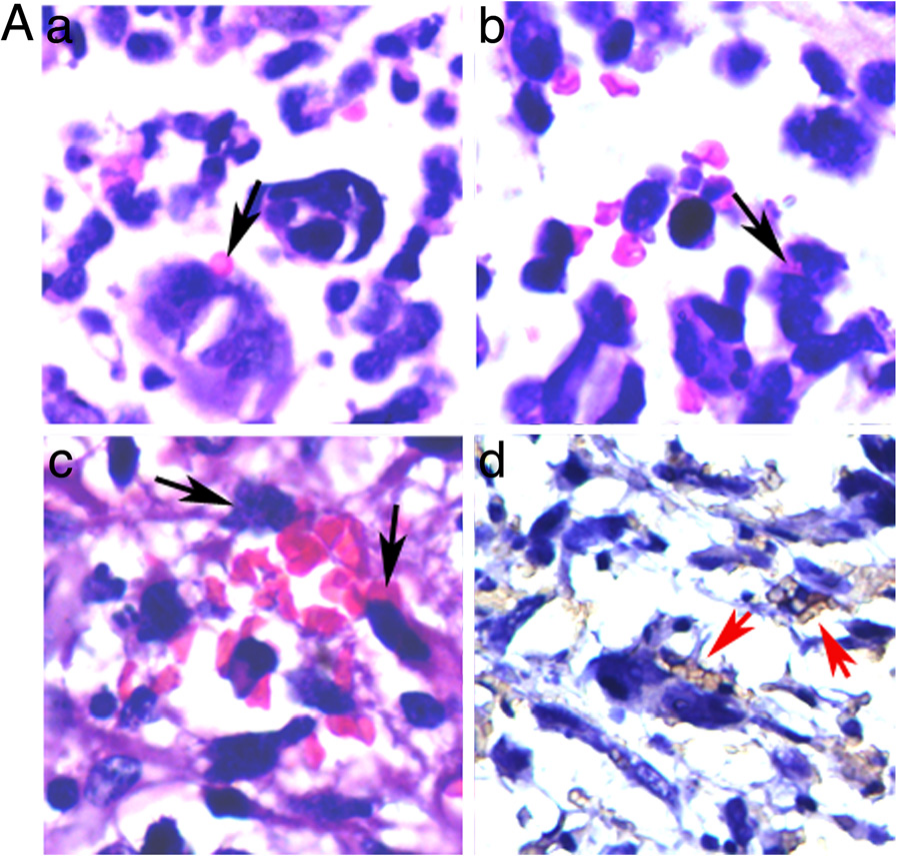
**Human high grade glioma cells generated erythrocytes. a)** H&E staining showed that there were many red bodies adhered to the surface of PGCCs (Black arrows point) (×200). **b)** Red bodies located in the cytoplasm of PGCC (Black arrows point) (×200). **c)** PGCCs and their budding erythrocytes form vessel-like structure with basement membrane (Black arrows point) (×200). **d)** IHC staining of hemoglobin-β/γ/ϵ/δ confirmed that the red bodies generated by PGCCs were erythrocytes (Red arrows point) (×200).

Zhang et al. reported that PGCCs of breast cancer cell line BT-549 was able to generate erythrocytes in vitro and in vivo [20]. To determine whether glioma PGCCs can directly generate erythrocytes, H&E and anti-hemoglobin-β/γ/ϵ/δ chain IHC staining were performed on glioma tissue sections and the results showed that there were many red bodies budding from PGCCs. These red bodies located in the cytoplasm or adhered to the surface of PGCCs (Figure 2A -a). Figure 2A-b showed that some red bodies located in the cytoplasm of PGCC. An interesting phenomenon indicated that some PGCCs generating erythrocytes form the wall of VM and MVs. Figure 2A-c showed that PGCCs and their generating erythrocytes can form VM structure and PGCCs lined in the basement membrane of VM. Hemoglobin-β/γ/ϵ/Δ IHC staining confirmed that these red bodies generated by PGCCs were erythrocytes (Figure 2A -d).

### Different patterns of blood supply in glioma tissues

**Figure 3:**
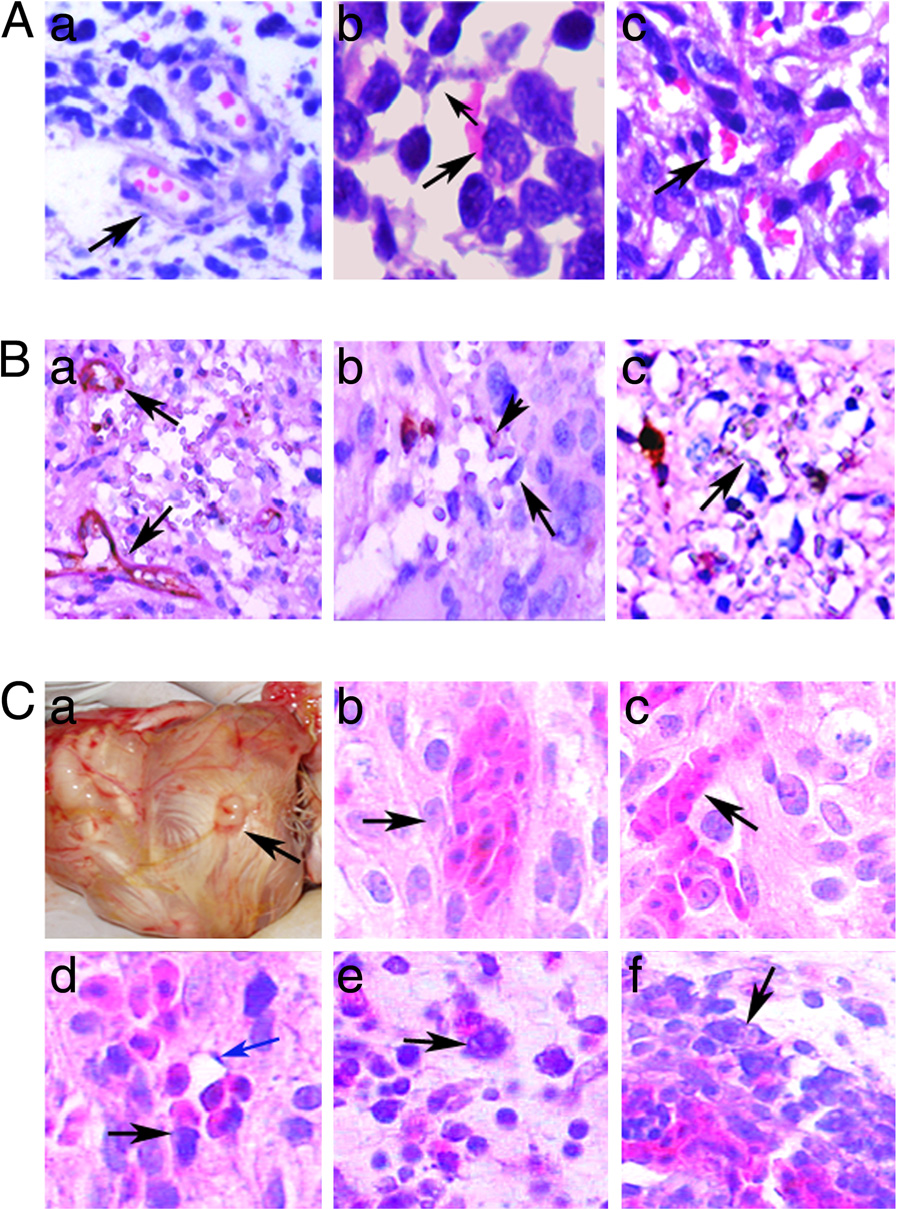
**Different blood supply patterns in human glioma tissues and C6 glioma cell xenografts. A**. Different blood supply patterns including EVs, MVs and VM in human gliomas. **a)** EVs in high grade gliomas (Black arrows point) (H&E × 200). **b)** Tumor cells (Large black arrow points) and endothelial cells (Small black arrow points) formed the structure of MV with red blood cells in it (H&E, ×400). **c)** VM in human high grade gliomas. Tumor cells formed the wall of VM (Black arrow points) with red blood cells in it (H&E, ×200). **B**. Double staining with CD31 IHC staining and PAS histochemical staining confirmed the wall structures of EVs, MVs and VM in human high grade gliomas. **a)** EVs were positive both for CD31 and PAS staining (Black arrows point) (×200). **b)** Tumor cells (CD31 negative staining, large black arrow points) and endothelial cells (CD31 positive staining, small black arrow points) formed the MV (×200). **c)** The wall of VM (black arrow points) was negative for CD31 staining and positive for PAS staining (×200). **C**. MVs, VM and PGCCs in human glioma cancer cell line C6 xenograft of chicken embryonating eggs. **a)** The gross imagine of the embryonating egg xenograft model (Black arrow point the tumor mass). **b** and **c)** VM in C6 xenografts with nucleated red blood cells in it (Black arrows point) (HE,×200). **d)** Tumor cells (Black arrow points) and endothelial cells (Blue arrow points) formed the structure of MVs with nucleated red blood cells in it (H&E, ×200). **e** and **f)** Presence of PGCCs in the embryonating eggs xenografts (Black arrows point) (H&E, ×200).

Different microcirculation patterns including VM, MVs and EVs appeared in glioma tissues with different grades. Figure 3A showed the different microcirculation patterns in glioma sections with H&E staining. Typical EVs were made of endothelial cells and basement membrane (Figure3A -a). Some PGCCs generating erythrocytes formed the wall of MVs (Figure 3A -b) and VM (Figure 3A -c). To further confirm the structure of different microcirculation patterns in gliomas, the sections were double-stained with endothelial cell-specific marker CD31 and PAS (basement membrane is positive for PAS staining). VM was identified by the presence of red blood cells in vessels lined by tumor cells, not by endothelial cells. As shown in Figure 3B, the wall of EVs was both positive for CD31 and PAS staining (Figure 3B-a). A single cell was positive for CD31 staining and the other cells were negative for MVs wall (Figure 3B-b). The wall of VM was negative for CD31 and PAS staining (Figure 3B-c). The average of VM counting in low grade and high grade gliomas was 0.7 ± 0.675 and 4.1 ± 0.994, respectively. There were more VM in high grade gliomas than that in low grade gliomas and the differences was statistically significant (Table 1). The wall of MVs was lined by both tumor and endothelial cells and there were more MVs in high grade gliomas than that in low grade gliomas (*t* = 4.789, *P* = 0.000; Table 1).

**Table 1:** The average number of VM, MVs and EVs in high and low grade human glioma

	**Low grade**	**High grade**	_**t**_	***p *** **value**
	**(I & II, n = 28)**	**(III & IV, n = 48)**		
VM	0.7 ± 0.675	4.1 ± 0.994	3.745	0.000
MVs	0.4 ± 0.516	2.6 ± 0.966	4.789	0.000
EVs	10.4 ± 3.03	14.7 ± 3.47	5.984	0.043

*VM*, vasculogenic mimicry; *MVs*, mosaic vessels; *EVs*, endothelium-dependent vessels.

### Presence of PGCCs, VM and MVs in chicken embryonating eggs with C6 xenografts

Different circulation patterns were further confirmed in chicken embronating eggs with C6 xenografts because of the nucleated red blood cells in chicken. We generated the xenografts in the chicken embryonating eggs with glioma C6 cell (Figure 3C -a). These xenografts were fixed with formalin. H&E staining data showed that VM appeared in the xenografts with nucleated red blood cells in it (Figure 3C –b and -c). Furthermore, MVs formed by endothelial and tumor cells occurred in C6 xenografts with nucleated red blood cells in the channels of MVs (Figure 3C -d). PGCCs can also be observed in glioma cell C6 xenografts (Figure 3C –e and -f).

## Discussion

Glioma is a type of tumor that occurs in the brain or spine. Glioma makes up to 30% of all brain and central nervous system tumors and 80% of all malignant brain tumors [26,27]. Glioma can be categorized according to their grade, which is determined by pathologic evaluation of the tumor. Low grade glioma is well-differentiated, more benign with better prognosis [28]. Low grade gliomas grow slowly, often over many years, and undergo surgery or not based on the locations and symptoms. However, high grade glioma is more undifferentiated and malignant with poor prognosis [29]. Morphologic characteristics and proliferation rate which indicate by Ki-67 IHC staining are the basis of the glioma grading [30,31]. The Ki-67 protein is a cellular marker for proliferation [32,33] and often used to assess the glioma grade [31,34].

Extensive areas of necrosis often appear in high grade glioma, which indicate the hypoxic microenvironment in tumor. The normal response to hypoxia is to stimulate the growth of new blood vessels and other blood supply patterns. Tumor hypoxia is well recognized as a major driving factor related with many tumor biological behaviors and associated with the formation and maintenance of cancer stem cells [35,36]. Previous studies showed that hypoxia can promote the self-renewal capability of the stem and non-stem cell population as well as promoting stem-like phenotype expression in the non-stem population and tumorigenesis [37]. Hypoxia can prevent the differentiation of neural stem cells in vitro [38]. PGCCs is an important heterogeneity of solid human cancers [1,2] and Zhang et al. reported that PGCCs had the properties of cancer stem cell and could be induced by hypoxic condition [11]. PGCCs are the most commonly described histopathology features of human tumors, particularly in high grade and advanced stage of the disease and thus, usually correlate with poor prognosis [3-5]. High grade glioma is typically heterogeneous [39]. Results of our current study confirmed that there were more PGCCs in high grade gliomas than those in the low grade gliomas, which may indicate that the number of PGCCs associated with hypoxia condition in high grade gliomas. Furthermore, most of the PGCCs located around the necrotic areas and the boundary between normal and tumor tissue. The hypoxic microenvironment around the necrosis induced the formation of PGCCs. In the boundary, tumor cells need sufficient oxygen and nutrient to form the “infiltration striker” invading into the normal tissue. The “relative” hypoxia can also induce the formation of PGCCs.

Tumor cells can express angiogenesis factors and recruit normal endothelial cells to form neoangiogenesis to support tumor proliferation and expansion. Neoangiogenesis is a well-established mechanism that sustains the aggressive growth of high-grade tumors [40-42]. VM and MVs are independent of traditional angiogenesis. The wall of VM is lined by tumor cells and/or basement membrane, and no endothelial cells are found on its inner wall. MV is another type of pattern, where the wall of MVs is lined both endothelial cells and tumor cells randomly. Red blood cells can flow through VM and MVs [2]. The number of VM and MVs were also associated with tumor grade, invasion and metastasis. In this study, we provided evidences that the number of VM and MVs were associated with the grade in gliomas. High grade glioma has extensive areas of necrosis, where the hypoxic microenvironment can stimulate the formation of new blood supply patterns besides PGCCs formation.

In the beginning of this study, we unexpectedly found many red bodies located in the cytoplasm or around the PGCCs, which form the structures of VM and MVs. IHC staining confirmed that these red bodies were positive for hemoglobin-β/γ/ϵ/δ. These red bodies were neither red blood cells derived from the hemorrhage, which there is diffuse red blood cells distribution during the process of hemorrhage, nor russell bodies which were homogenous immunoglobulin. Zhang et al. reported that many kinds of cancer cell line were able to directly generate hemoglobin and erythrocytes both in vitro and in vivo using hypoxia mimic CoCl_2_[20]. VM was first reported by Maniotist in 1999 [43]. However, the detailed process of VM formation and origin of erythrocytes is still unclear. Since tumor cells can generate erythrocytes, we can infer that tumor cells and their generating erythrocytes can form VM or MVs structure in high grade tumor. Our data provided a novel concept to understand VM formation though the current study is just a proof-of-principle. However, most of experimental data in our study are descriptive and the detailed molecular mechanisms need to be provided in the future.

## Conclusions

The number of PGCCs, VM and MVs increased with the malignant grade in gliomas. PGCCs generated erythrocytes to form VM and MVs.

## Abbreviations

PGCCs: Polyploid giant cancer cells; IHC staining: Immunohistochemical staining; VM: Vasculogenic mimicry; MV: Mosaic vessel, EV, endothelium dependent vessel. CD31, the platelet–endothelial cell adhesive molecule; PAS: Periodic acid–Schiff.

## Competing interests

The authors declare that they have no competing interests.

## Authors’ contributions

QY and ZL: collection and/or assembly of data, conception and design, manuscript writing. RZ and HT: data analysis and interpretation. ZS: conception and design, financial support, manuscript writing; final approval of manuscript. All authors read and approved the final manuscript.
